# A large *de novo* 9p21.3 deletion in a girl affected by astrocytoma and multiple melanoma

**DOI:** 10.1186/1471-2350-15-59

**Published:** 2014-05-17

**Authors:** Simona Frigerio, Vittoria Disciglio, Siranoush Manoukian, Bernard Peissel, Gabriella Della Torre, Andrea Maurichi, Paola Collini, Barbara Pasini, Giacomo Gotti, Andrea Ferrari, Licia Rivoltini, Maura Massimino, Monica Rodolfo

**Affiliations:** 1 Department of Experimental Oncology and Molecular Medicine, Fondazione IRCCS Istituto Nazionale dei Tumori, via Venezian 1, Milan 20133, Italy; 2 Department of Experimental Oncology and Molecular Medicine, Functional Genomics and Bioinformatics Core Facility, Fondazione IRCCS Istituto Nazionale dei Tumori, via Venezian 1, Milan 20133, Italy; 3 Unit of Medical Genetics, Department of Preventive and Predictive Medicine, Fondazione IRCCS Istituto Nazionale dei Tumori, Milan, Italy; 4 Unit of Melanoma and Sarcoma, Department of Surgery, Fondazione IRCCS Istituto Nazionale dei Tumori, Milan, Italy; 5 Department of Pathology, Fondazione IRCCS Istituto Nazionale dei Tumori, Milan, Italy; 6 Current address: Department of Medical Science, University of Turin, Turin, Italy; 7 Pediatric Unit, Fondazione IRCCS Istituto Nazionale dei Tumori, Milan, Italy

**Keywords:** Melanoma-astrocytoma syndrome, 9p21.3 deletion, *CDKN2A*, *CDKN2BAS*, MLPA, Oligo array-CGH

## Abstract

**Background:**

Association of melanoma, neural system tumors and germ line mutations at the 9p21 region in the *CDKN2A*, *CDKN2B* and *CDKN2BAS* genes has been reported in a small number of families worldwide and described as a discrete syndrome in melanoma families registered as a rare disease, the melanoma–astrocytoma syndrome.

**Case presentation:**

We here studied two young patients developing melanoma after radiotherapy for astrocytoma, both reporting lack of family history for melanoma or neural system tumors at genetic counselling. Patient A is a girl treated for anaplastic astrocytoma at 10 years and for multiple melanomas on the scalp associated to dysplastic nevi two years later. Her monozygotic twin sister carried dysplastic nevi and a slow growing, untreated cerebral lesion. Direct sequencing analysis showed no alterations in melanoma susceptibility genes including *CDKN2A*, *CDK4*, *MC1R* and *MITF* or in *TP53*. By microsatellite analysis, multiplex ligation-dependent probe amplification, and array comparative genomic hybridization a deletion including the *CDKN2A, CDKN2B* and *CDKN2BAS* gene cluster was detected in both twin sisters, encompassing a large region at 9p21.3 and occurring *de novo* after the loss of one paternal allele.

Patient B is a boy of 7 years when treated for astrocytoma then developing melanoma associated to congenital nevi on the head 10 years later: sequencing and multiplex ligation-dependent probe amplification revealed a normal profile of the *CDKN2A/CDKN2B/CDKN2BAS* region. Array comparative genomic hybridization confirmed the absence of deletions at 9p21.3 and failed to reveal known pathogenic copy number variations.

**Conclusions:**

By comparison with the other germ line deletions at the *CDKN2A, CDKN2B* and *CDKN2BAS* gene cluster reported in melanoma susceptible families, the deletion detected in the two sisters is peculiar for its *de novo* origin and for its extension, as it represents the largest constitutive deletion at 9p21.3 region identified so far.

In addition, the two studied cases add to other evidence indicating association of melanoma with exposure to ionizing radiation and with second neoplasm after childhood cancer. Melanoma should be considered in the monitoring of pigmented lesions in young cancer patients.

## Background

The combination of melanoma and neural system tumors (NST) was described in melanoma families where the diseases occurred in different family members or concomitantly, and was registered as a rare disease, the melanoma-astrocytoma syndrome (OMIM 155755).

Three families from France, UK and USA showing association of melanoma, NST and germ line deletions of part or of the entire *CDKN2A* locus, as well as of *CDKN2B* and the non-coding *CDKN2BAS* genes, have been described [1-3]. In other studies, families prone to melanoma and NST lacked deletions in the 9p21 region, although other altered loci causing the syndrome have not been identified [4-7]. Missense mutations in *CDKN2A* genes were also described in one French and two Italian families reporting melanoma associated with meningioma and neuroblastoma respectively [8,9]. The analysis of other genes located in this region, such as *KLHL9* and *PTPRD*, failed to identify inherited mutations in melanoma and NST kindreds [7,10].

While the genetic basis for NST remains largely unidentified [11], several genes associated to melanoma predisposition besides *CDKN2A* have been recently identified [12].

In a recent study we genetically characterized a series of 21 pediatric melanoma treated at our Institute [13]: two cases developing melanoma after NST were studied for deletions at the 9p21 region. Here we report the results of these analyses.

## Case presentation

**Figure 1:**
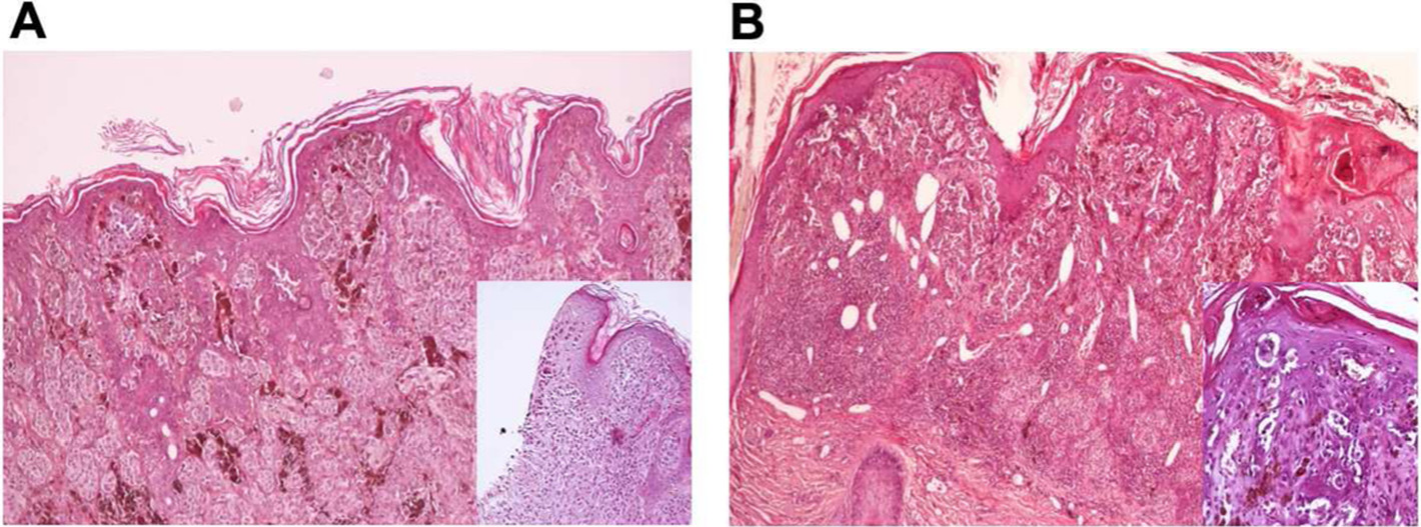
**Histopathology of the initial biopsy specimens excised from the head of patient A.** Specimens from the head neoplasms of the patient revealing a superficial spreading melanoma with epithelioid appearance associated with a compound nevus (inset) **(A)** or in the absence of melanocytic nevi **(B)**. Hematoxylin and eosin, magnification 10×.

We report the case of a female Italian patient (A) treated for anaplastic astrocytoma (10 yr) who developed multiple melanomas on the scalp associated to dysplastic nevi two years later (Figure 1). In the following 8 years when she was followed clinically at our Institute, she developed 10 melanomas on the head, neck, trunk and leg. She also developed neurotechoma (8 yr) and neurofibroma (18 yr). A tectal mesencephalic lesion growing along 10 years and producing hydrocephalus was the final reason for her death (20 yr). Her monozygotic twin sister (TS) carried dysplastic nevi and a slow growing, untreated cerebral lesion at parietal cortex (22 yr). The pedigree profile at genetic counselling lacked neoplastic diseases in the maternal lineage while in the paternal lineage an uncle and his son had unspecified neoplastic disease.

The sequencing analysis showed wild type sequences for *CDKN2A*, *CDK4*, and *MC1R;* in addition, *TP53* mutations and the *MITF* E318K variant identified in familial melanoma [14,15] were not found (data not shown) (see Additional file 1: Methods). The allele status of microsatellite markers in the *CDKN2A* gene region was analyzed in patient A, her parents and TS (see Additional file 2: Table S1). For the markers D9S974, D9S942, D9S1748 and D9S1749 a single peak was detected for the patient and the TS, while for the parents two alleles with at least one allele of the same size were shown. For each marker, the allele found had the same size of one of the maternal allele (see Additional file 3: Figure S1) indicating the occurrence of a deletion in the *CDKN2A* locus in the paternal allele.

**Figure 2:**
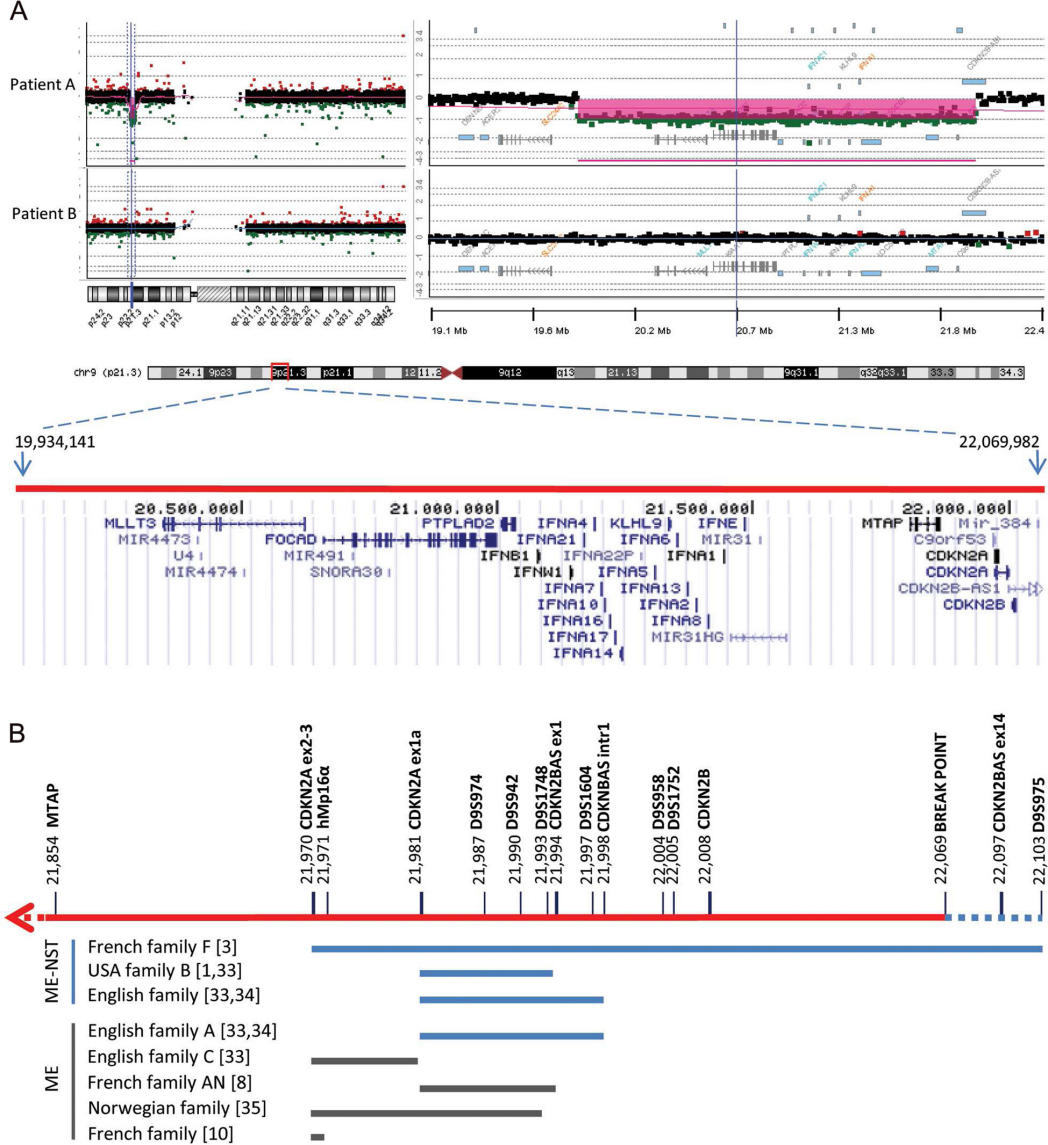
**Schematic representation of the deletion identified in patient A and of germ line deletions at the 9p21.3 region reported in other studies. A.** Array-CGH 400K ratio profile: on the left, the chromosome ideogram, on the right, the log2 ratio of chromosome 9 probes plotted as a function of chromosomal position. Probes with a value zero represent equal fluorescence intensity ratio between sample and reference DNAs; each filled rectangle represents a single probe spotted on the array. Copy number losses shift the ratio to negative log ratio values. In the lower part, the deleted region at 9p21.3 detected in patient A is shown in red and the deleted genes are represented according to UCSC Genome Browser (NCBI build 37, hg19). The deleted region spans between oligomers at 19,934,142 Mb (A_18_P26451569, first deleted) and 22,069,983 Mb (A_16_P18578628, last deleted), flanked by oligomers at 19,927,491 Mb (A_16_P18573439, first present) and 22,086,798 Mb (A_16_P18578677, last present). No CNVs are detected for patient B in the same region. **B.** Representation of the deletions previously reported in melanoma-NST (ME-NST) and in melanoma (ME) kindreds, in blue and grey lines respectively, in comparison to the deletion detected in patient A. The status of microsatellite markers and of genes comprised in the region as derived from MLPA is shown. The start nucleotide position for microsatellites and genes is indicated (UCSC Genome Browser) [16-18].

Multiplex Ligation-dependent Probe Amplification (MLPA) showed hemizygosity of *CDKN2A*-*CDKN2B* gene region for patient A and TS, and normal profiles for the parents (see Additional file 4: Figure S2), supporting a *de novo* origin. The analysis of metaphase chromosomes from EBV-immortalized lymphocytes by FISH hybridization using as probe the C5 cosmid spanning about 50 kb of the chromosomal region from *CDKN2A* to *CDKN2B* genes further confirmed the deletion in the two sisters (data not shown). Oligo array-comparative genomic hybridization (aCGH) analysis was performed to confirm MLPA results and to better define the deletion breakpoints. The analysis of ratio profiles revealed for patient A and TS a deletion at the 9p21.3 chromosomal region of approximately 2,135 Mb including part of the *CDKN2BAS* gene*, CDKN2B, CDKN2A, MTAP*, *MIR31*, the *IFNA* gene cluster*, KLHL9, IFNW1, IFNB1, PTPLAD2, FOCAD, MIR491, SNORA30, MLLT3*, *MIR4473* and *MIR4474* (Figure 2A).

The second case studied, patient B, is a boy developing melanoma associated to congenital nevi on the head (17 yr) after astrocytoma (7 yr). Five years later he developed fatal pleuric rhabdomyosarcoma. His pedigree lacked neoplastic diseases. No alterations were revealed by sequence analysis in *CDKN2A* gene except for the rs3814960 and rs11515 variants in the 3′UTR and 5′UTR regions*,* which are rather common single nucleotide polymorphisms (SNPs) in melanoma patients [19]. In addition, he carried wild type *CDK4*, *MC1R* and *MITF* genes, and a normal profile of the *CDKN2A-CDKN2B* region was shown by MLPA. aCGH analysis showed a normal ratio profile at the 9p21.3 region (Figure 2A) and revealed copy number gains on 4p14 and 6q24.3 of uncertain clinical significance, which deserve further studies and analyses in other patients with similar diseases (see Additional file 5: Table S2).

## Conclusions

Germ line mutations at *CDKN2A* locus are generally inherited founder mutations having a common ancestral origin, while de novo mutations appear to be exceedingly rare events [20], thus marking a peculiarity in the genetic of patient A. Although the precise endpoints of the deletion were not determined here, by aCGH it was shown to span from 19,934,142 to 22,069,983 Mb, thus being larger than those previously mapped by Pasmant [3]. In fact, in addition to the *CDKN2A/CDKN2B/CDKN2BAS* gene cluster, the deletion extends in the telomeric end to comprise a large region up to the *MLLT3* gene.

Similarly to the other deletions detected in association with melanoma and NST, the deleted region includes part of the *CDKN2BAS* gene, which on the contrary is not always included in the deletions occurring in melanoma kindreds lacking NST, as schematized in Figure 2B. *CDKN2BAS* gene, or *ANRIL* (antisense non-coding RNA in the *INK4* locus), consists of 19 exons spanning a region of 126.3 kb located within the *CDKN2B-CDKN2A* gene cluster, and is transcribed in the antisense orientation in a long non-coding RNA involved in epigenetic silencing of *CDKN2B-CDKN2A* locus by polycomb repressive complexes [21]. Genome-wide association studies have identified SNPs in *CDKN2BAS* associated with susceptibility to NST as well as to melanoma [22,23]; interestingly, *CDKN2BAS* has been identified as a risk locus also for other cancers and diseases [24]. Among the other genes comprised in the deleted region, *FOCAD* has been shown deleted in glioma tumors [25]. Furthermore, in patient B, CGH analysis showed copy number gains on 4p14 and 6q24.3 chromosomal regions involving *TCF25* and *KLF3* genes respectively, which encode for transcription factors, representing potential candidates for further studies. In fact, *TCF25*, has been involved in embryonic development expressed in brain [26], and *KLF3*, has been reported to show rearrangements in different cancer types [27].

Our study shows that 9p21.3 deletion is neither necessary nor sufficient for the NST-melanoma syndrome. Of note, both patients developed melanoma on their head after radiotherapy for astrocytoma, thus adding to other evidence suggesting association of melanoma with exposure to ionizing radiation [28-32]; in addition, the TS of patient A, who was not treated for NST, did not develop melanoma, although she carried an identical 9p21.3 deletion (see Additional file 6: Figure S3). It results that young cancer patients treated with radiotherapy should be considered at risk for developing melanoma and their pigmented lesions should be carefully monitored by expert clinicians. In fact, although melanoma is a rare disease in the healthy childhood population, it occurs more frequently as second malignant neoplasm after childhood cancer [33] and should be closely monitored by regularly screen in the follow-up of survivors, which have an approximate 2.5-fold increased risk of melanoma [34]. Furthermore, children with genetic syndromes may have unique pathophysiologies that necessitate careful evaluation and follow-up of skin alterations, since often dermatologists find unusual and atypical correspondences between clinic and dermoscopic parameters and the histological ones [35]. A multifaceted approach including a thorough clinical history, visual examination and dermoscopic evaluation of suspicious skin lesions is recommended to increase the sensitivity and specificity for diagnosing melanoma in these young patients.

## Consent

Written informed consent was obtained from the patients’ parents for genetic counselling, DNA analyses, scientific research and study purposes for all family members. The consent form was approved by the local ethical committee (Comitato Etico Centrale IRCCS Lombardia). A copy of the written consent is available for review by the Editor of this journal.

## Abbreviations

NST: Neural system tumor; MLPA: Multiplex ligation-dependent probe amplification; TS: Twin sister; SNP: Single nucleotide polymorphism; aCGH: array Comparative Genomic Hybridization; CNV: Copy number variation.

## Competing interests

The authors declare that they have no competing interests.

## Authors’ contributions

SF carried out the PCR amplifications, sequencing studies, MLPA and microsatellite analyses and helped to draft the manuscript. VD carried out array-CGH, the analysis of the results and helped to write the final manuscript. AM and PC carried out the clinical and the pathologic analyses and helped to draft the manuscript. SM, BP and PP the genetic counselling and helped to draft the manuscript. GDT designed the study and the genetic analysis. GG, AF and MM conceived of the study, and participated in its design and coordination and helped to draft the manuscript. MR and LR designed the study, defined the results and finalized the manuscript. All authors read and approved the final manuscript.

## Pre-publication history

The pre-publication history for this paper can be accessed here:

http://www.biomedcentral.com/1471-2350/15/59/prepub

## Supplementary Material

Additional file 1 Methods. Click here for file

Additional file 2: Table S1 Primers of the microsatellite markers used in the study. Start and end positions are reported according to UCSC Genome Browser (NCBI Build 37, hg19). Click here for file

Additional file 3: Figure S1 Microsatellite analysis revealing loss of the paternal allele in patient A and TS. Homozygosity or hemizygosity at microsatellite loci was analyzed by PCR and amplification products analyzed with the ABI Prism Peak Scanner Software. Click here for file

Additional file 4: Figure S2 Results of MLPA of the 9p region in patient A, TS and parents. Results obtained with MLPA arrays for the 9p21 region ordered according to the genomic location of the probes. Gene dosage quotients for the 41 probes and relative ID numbers are shown, for patient A and TS in full bars, and for parents in empty bars. The deletion detected in the two sisters extends from *CDKN2B* to *MLLT3* genes, and includes *CDKN2A, MTAP, IFNA1, KLH9, IFNW1,* and *IFNB1* genes; in contrast, *ELAV2* and *TEK* centromeric to *CDKN2B* and *GLDC* and *DOCK8* telomeric to *MLLT3* showed normal gene dosage quotients indicating retention of both gene copies. Click here for file

Additional file 5: Table S2 List of chromosomal aberrations detected by aCGH (400K) in patient B not reported in the Database of Genomic Variants (http://dgv.tcag.ca/dgv/app/home?ref=GRCh37/hg19). CNVs in non coding regions were detected on 7p12.3, 7q11.22, 19q13.11 chromosomal regions. A large region on 14q11.2 contains genes poorly characterize functionally. CNVs on 4p14 and 16q24.3 involve genes of potential interest since they have been involved in the regulation of cell growth and death. Click here for file

Additional file 6: Figure S3 Results of array-CGH of the 9p21.3 region in patient A and her twin sister. aCGH analysis showed an identical 9p21.3 deletion of _~_ 2,135 Mb. Click here for file
