# Supplementary material for: A large de novo 9p21.3 deletion in a girl affected by astrocytoma and multiple melanoma
Source: BMC Med Genet. 2014 May 17;15:59. doi: 10.1186/1471-2350-15-59 (PMC4036080; doi:10.1186/1471-2350-15-59)
Supplement: Additional file 1 — Methods. [file 1471-2350-15-59-S1.pdf]

Supplemental Methods

Sequencing. Genomic DNA was extracted from peripheral blood using standard procedures and its quality was monitored using a Nanodrop spectrophotometer. DNA was amplified for CDKN2A, CDK4, MC1R, TP53 and MITF as described previously [13, S1]. PCR products were subjected to automatic sequencing by ABI PRISM 310 genetic analyzer (Applied Biosystems).

Microsatellite analysis. The primers used for microsatellite typing are reported in Table S1. In each primer pair, a 5'-FAM oligonucleotide was used. Amplification products were visualized as described for Sequencing. Analysis was conducted using Peak Scanner Software v1.0 using GeneScan-400 TAMRA size standard (Applied Biosystems).

Multiplex ligation-dependent probe amplification (MLPA). MLPA kits ME024-B1-salsa and P024-B2-salsa were used to profile the 9p21 region as detailed by the manufacturer instructions (MRC-Holland, Amsterdam, the Netherlands). Probe amplification products were run on an ABI PRISM 310 genetic analyzer using GeneScan-500 TAMRA size standard. Results were analyzed by Coffalyser v9.4 software after normalization of peaks against three samples of DNA from healthy donors.

Array-CGH. The array-CGH analysis was performed using the SurePrint G3 Human CGH Agilent 2x400K array as recommended by Agilent (protocol Version 7.2 July 2012 Agilent Technologies). A graphical visualization of the results was provided by the Agilent CytoGenomics (v 2.7.22.0) with the following parameters: aberration algorithm ADM-2, threshold 6.0 and moving average window 1Mb. Aberrant signals including 3 or more consecutive probes with the same polarity were called as deletion or duplication. Physical

mapping and gene location were obtained from the UCSC Genome Browser (hg19, NCBI Build 37).

Supplemental References

S1. Pollio A, Tomasi A, Seidenari S, Pellacani G, Rodolfo M, Frigerio S, Maurichi A, Turchetti D, Bassoli S, Ruini C, Ponti G. **Malignant and benign tumors associated with multiple primary melanomas: just the starting block for the involvement of MITF, PTEN and CDKN2A in multiple cancerogenesis?** *Pigment Cell Melanoma Res* 2013, **26**:755-757.

S2. Presti JC Jr, Wilhelm M, Reuter V, Russo P, Motzer R, Waldman F. **Allelic loss on chromosomes 8 and 9 correlates with clinical outcome in locally advanced clear cell carcinoma of the kidney.** *J Urol* 2002,**167**:1464-1468.

S3. Grady B, Goharderakhshan R, Chang J, Ribeiro-Filho LA, Perinchery G, Franks J, Presti J, Carroll P, Dahiya R. **Frequently deleted loci on chromosome 9 may harbor several tumor suppressor genes in human renal cell carcinoma**. *J Urol* 2001,**166**:1088-1092.

S4. Smeds J, Kumar R, Rozell BL, Hemminki K. **Increased frequency of LOH on chromosome 9 in sporadic primary melanomas is associated with increased patient age at diagnosis.** *Mutagenesis* 2000,**15**:257-260.

S5. Pollock PM, Spurr N, Bishop T, Newton-Bishop J, Gruis N, van der Velden PA, Goldstein AM, Tucker MA, Foulkes WD, Barnhill R, Haber D, Fountain J, Hayward NK. **Haplotype analysis of two recurrent CDKN2A mutations in 10 melanoma families: evidence for common founders and independent mutations.** *Human Mutation* 1998,**11**:424-431.

S6. Mistry SH, Taylor C, Randerson-Moor JA, Harland M, Turner F, Barrett JH, Whitaker L, Jenkins RB, Knowles MA, Bishop JA,Bishop DT. **Prevalence of 9p21 deletions in UK melanoma families.** *Genes Chromosomes Cancer* 2005,**44**:292-300.
