# Supplementary material for: A large de novo 9p21.3 deletion in a girl affected by astrocytoma and multiple melanoma
Source: BMC Med Genet. 2014 May 17;15:59. doi: 10.1186/1471-2350-15-59 (PMC4036080; doi:10.1186/1471-2350-15-59)
Supplement: Additional file 2: Table S1 — Primers of the microsatellite markers used in the study. Start and end positions are reported according to UCSC Genome Browser (NCBI Build 37, hg19). [file 1471-2350-15-59-S2.pdf]

Table S1. Primers of the microsatellite markers used in the study. Start and end positions are reported according to UCSC Genome Browser (hg19, NCBI Build 37).

microsatellite	FORWARD PRIMER	REVERSE PRIMER	start	end	ref
D9S921	GGTAAGTCAGCTATAATGATC	CTCTTTCATGTTGGCTCCTGT	10509506	10509623	S2
D9S1839	CTTGTTTATCTCAAGGAAATACGTG	CCATAGTCAGTGTGCGCT	16418749	16419098	S3
D9S157	AGCAAGGCAAGCCACATTTC	TGGGGATGCCCAGATAACTATATC	17628219	17628591	S3
D9S162	GCAATGACCAGTTAAGGTTC	AATTCCCACAACAAATCTCC	19679803	19680020	S3
D9S736	TTCTAGACCTCTCAGCAGAC	GATAGTGTTGGAGACACCAG	21636294	21636463	S4
D9S974	GAGCCTGGTCTGGATCATAA	AAGCCTACAGAACCAGACAG	21987353	21987560	S5
D9S942	GCAAGATTCCAAACAGTA	CTCATCCTGCGGAAACCATT	21990577	21990675	S5
D9S1748	CACCTCAGAAGTCAGTGAGT	GTGCTTGAAATACACCTTTCC	21993775	21993894	S5
D9S171	AGCTAAGTGAACCTCATCTCTGTCT	ACCCTAGCACTGATGGTATAGTCT	24534209	24534427	S6
