# Supplementary material for: A large de novo 9p21.3 deletion in a girl affected by astrocytoma and multiple melanoma
Source: BMC Med Genet. 2014 May 17;15:59. doi: 10.1186/1471-2350-15-59 (PMC4036080; doi:10.1186/1471-2350-15-59)
Supplement: Additional file 3: Figure S1 — Microsatellite analysis revealing loss of the paternal allele in patient A and TS. Homozygosity or hemizygosity at microsatellite loci was analyzed by PCR and amplification products analyzed with the ABI Prism Peak Scanner Software. [file 1471-2350-15-59-S3.pdf]

Figure S1: Microsatellite analysis revealing loss of the paternal allele in patient A and TS. Homozygosity or hemizygosity at microsatellite loci was analyzed by PCR and amplification products analyzed with the ABI Prism Peak Scanner Software.

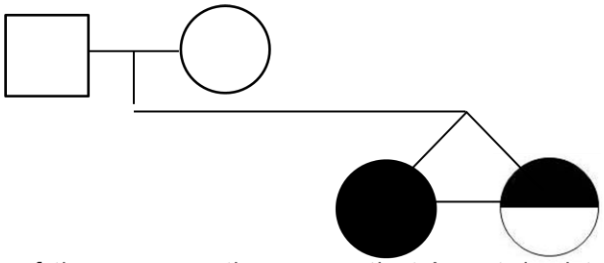

	markers	father	mother	patient A	twin sister
9p tel	D9S921	80	83 97	80 97	80 97
	D9S1839	122	122	122	122
	D9S157	194 206	196 206	194 196	194 196
	D9S162	180	170 180	170 180	170 180
	D9S736	170	166 170	170	170
	D9S974	203 211	205	205	205
	D9S942	112 116	118	118	118
	D9S1748	106	110 118	110	110
9p cent	D9S171	162 172	168 170	162 170	162 170
