# Supplementary material for: A large de novo 9p21.3 deletion in a girl affected by astrocytoma and multiple melanoma
Source: BMC Med Genet. 2014 May 17;15:59. doi: 10.1186/1471-2350-15-59 (PMC4036080; doi:10.1186/1471-2350-15-59)

Figure S2. Results of MLPA of the 9p region in patient A, TS and parents. Results obtained with MLPA arrays for the 9p21 region were ordered according to the genomic location of the probes. Gene dosage quotients for the 41 probes and relative ID numbers are shown, for patient A and TS in full bars, and for parents in empty bars. The deletion detected in the two sisters extends from *CDKN2B* to *MLLT3* genes, and includes *CDKN2A, MTAP, IFNA1, KLH9, IFNW1,* and *IFNB1* genes; in contrast, *ELAV2* and *TEK* centromeric to *CDKN2B* and *GLDC* and *DOCK8* telomeric to *MLLT3* showed normal gene dosage quotients indicating retention of both gene copies.

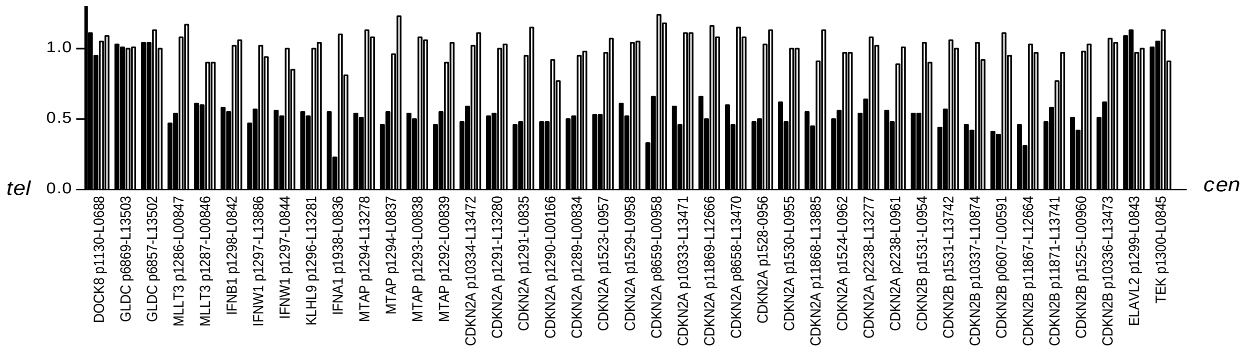

Supplement: Additional file 4: Figure S2 — Results of MLPA of the 9p region in patient A, TS and parents. Results obtained with MLPA arrays for the 9p21 region ordered according to the genomic location of the probes. Gene dosage quotients for the 41 probes and relative ID numbers are shown, for patient A and TS in full bars, and for parents in empty bars. The deletion detected in the two sisters extends from CDKN2B to MLLT3 genes, and includes CDKN2A, MTAP, IFNA1, KLH9, IFNW1, and IFNB1 genes; in contrast, ELAV2 and TEK centromeric to CDKN2B and GLDC and DOCK8 telomeric to MLLT3 showed normal gene dosage quotients indicating retention of both gene copies. [file 1471-2350-15-59-S4.pdf]
