# Supplementary material for: A large de novo 9p21.3 deletion in a girl affected by astrocytoma and multiple melanoma
Source: BMC Med Genet. 2014 May 17;15:59. doi: 10.1186/1471-2350-15-59 (PMC4036080; doi:10.1186/1471-2350-15-59)
Supplement: Additional file 5: Table S2 — List of chromosomal aberrations detected by aCGH (400K) in patient B not reported in the Database of Genomic Variants (http://dgv.tcag.ca/dgv/app/home?ref=GRCh37/hg19). CNVs in non coding regions were detected on 7p12.3, 7q11.22, 19q13.11 chromosomal regions. A large region on 14q11.2 contains genes poorly characterize functionally. CNVs on 4p14 and 16q24.3 involve genes of potential interest since they have been involved in the regulation of cell growth and death. [file 1471-2350-15-59-S5.pdf]

Table S2. List of chromosomal aberrations detected by aCGH (400K) in patient B not reported in the Database of Genomic Variants (http://dgv.tcag.ca/dgv/app/home?ref=GRCh37/hg19). CNVs in non coding regions were detected on 7p12.3, 7q11.22, 19q13.11 chromosomal regions. A large region on 14q11.2 contains genes poorly characterized functionally. CNVs on 4p14 and 16q24.3 involve genes of potential interest since they have been involved in the regulation of cell growth and death.

**Chromosomal Region**	**Start**	**End**	**Probes**	**Size (Kb)**	**Type**	**Gene Content**	**Gene Function**
4p14	38678882	38710915	8	32,03	DUP	*KLF3*	*Kruppel-Like Factor 3:* belong to Sp/KLF family of transcription factors. It has a characteristic zinc finger domain, but lack the N-terminal buttonhead box found in related SP transcription factors.
7p12.3	46700759	46723906	3	23,14	DUP		
7q11.22	70332231	70356011	3	23,78	DUP		
14q11.2	19376762	20420790	35	1044	DUP	AK022914	No protein
						LINC00516	No protein
						BC017398	No protein
						P775P	No protein
						DQ573684	No protein
						DQ595048	No protein
						DQ573684	No protein
						DQ582484	No protein
						POTEM	*POTE ankyrin domain family, member M*
						P712P	No protein
						OR11H, OR4Q3, OR4M, OR4N2, OR4K2, OR4K5, OR4K1	*Olfactory receptors* proteins that interact with odorant molecules in the nose, to initiate a neuronal response that triggers the perception of a smell. OR are members of a large family of G receptors (GPCR) arising from single coding-exon genes. The family is the largest in the human genome.
16q24.3	89956932	89981670	5	24,74	DUP	*TCF25*	*Transcription factor 25 (basic helix-loop-helix)* is a member of the basic helix-loop-helix (bHLH) family of transcription factors that are important in embryonic development.
19q13.11	35852152	35861485	3	9,334	DUP		
