# Supplementary material for: A large de novo 9p21.3 deletion in a girl affected by astrocytoma and multiple melanoma
Source: BMC Med Genet. 2014 May 17;15:59. doi: 10.1186/1471-2350-15-59 (PMC4036080; doi:10.1186/1471-2350-15-59)

Figure S3. Results of array-CGH of the 9p21.3 region in patient A and her twin sister. aCGH analysis showed an identical 9p21.3 deletion of ~ 2,135Mb.

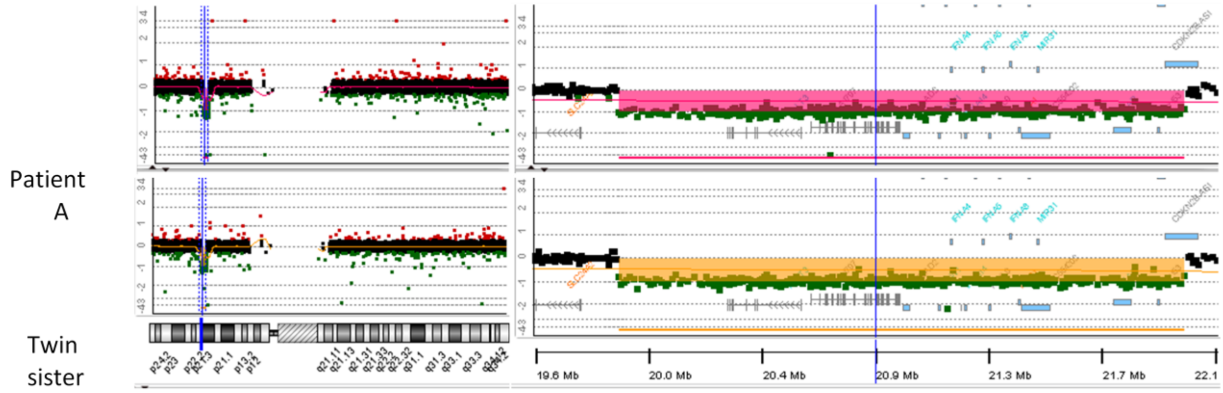

Supplement: Additional file 6: Figure S3 — Results of array-CGH of the 9p21.3 region in patient A and her twin sister. aCGH analysis showed an identical 9p21.3 deletion of ~ 2,135 Mb. [file 1471-2350-15-59-S6.pdf]
